# Disseminated Tuberculosis in a Healthy Adolescent Female

**DOI:** 10.7759/cureus.4967

**Published:** 2019-06-21

**Authors:** Amanda H Rosenthal, Lindsay D Rothfield, Laura Chamorro Dauer

**Affiliations:** 1 Pediatrics, University of Miami Miller School of Medicine, Miami, USA

**Keywords:** disseminated, tuberculosis, adolescent, healthy, female, puberty, hormones, hormonal, pubertal, immunocompetent

## Abstract

Disseminated tuberculosis (TB) is an uncommon sequela of Mycobacterium TB infection in which bacteria disseminate and form colonies outside of the lungs. Most reports of disseminated TB are found in immunocompromised patients, particularly in patients with human immunodeficiency virus (HIV) infection, malnutrition, genetic susceptibility, diabetes mellitus, smoking, and alcohol abuse. Few case reports describe the finding of disseminated TB in immunocompetent patients, especially in healthy adolescents. The purpose of this report is to present a case in which disseminated TB was found in an otherwise healthy adolescent, female patient, and to explore the effects of pubertal hormonal changes on the immune system. Several studies in the pediatric population have suggested that hormonal changes of estrogen and testosterone associated with puberty may influence the pathogenesis of active TB. While the exact pathogenesis of disseminated TB remains unknown, this case highlights the need for heightened awareness of TB among otherwise healthy adolescents, and specifically, the effect puberty has on disease progression.

## Introduction

Disseminated tuberculosis (TB), also known as miliary TB, is an uncommon sequela of Mycobacterium TB infection in which bacteria disseminate and form colonies outside of the lungs. The disseminated disease can lead to catastrophic results, such as meningitis, adrenal insufficiency, hepatic failure, pancreatic insufficiency, and splenic enlargement. Estimated mortality for patients suffering from disseminated TB is approximately 15%-20% in children and 25%-30% in adults, commonly due to delayed diagnosis. Early initiation of anti-TB medications has been shown to improve mortality [1]. Precise data on the incidence of disseminated TB is difficult to record due to frequent underreporting in developing nations. Based on the available data, most reports of disseminated TB are found in immunocompromised patients, particularly in patients with human immunodeficiency virus (HIV) infection, malnutrition, genetic susceptibility, diabetes mellitus, smoking, and alcohol abuse [1-2]. Few case reports describe the finding of disseminated TB in immunocompetent patients, specifically in healthy adolescents. In those studies that have focused on this specific population, it has been proposed that individuals in their pubertal years are at a higher risk for active TB than younger children, despite lack of additional risk factors [3]. These findings have suggested that hormonal changes during puberty create an environment within the host that allows for easy colonization and spread of Mycobacterium TB.

The purpose of this report is to present a case in which disseminated TB was found in an otherwise healthy adolescent female and to explore the effects of pubertal hormonal changes on the immune system which may have brought on the disseminated disease.

## Case presentation

A 16-year-old girl with no past medical history presented to an outside hospital’s emergency department because of an unintentional 9 kilogram (kg) weight loss over the past three months and a dry cough unresponsive to over-the-counter cough suppressants. The patient was born and raised in Spain and immigrated to the United States through Guatemala ten years ago with her mother and brother. She also endorsed a three-month history of amenorrhea. The patient denied hemoptysis, fevers, night sweats, sick contacts or travel outside of the United States since immigration.

On presentation, she was febrile (102.8F) and tachypneic (56 breaths per minute) with a normal oxygen saturation (SpO2) on room air. Physical exam was significant for failure to thrive with a body weight of 24 kg and height of 1.3 meters. She exhibited decreased breath sounds bilaterally and scattered rhonchi throughout all lung fields. Chest X-ray demonstrated extensive bilateral nodular opacities with a cavitary lesion in the upper right lung. Chest computed tomography (CT) showed extensive nodules and consolidations throughout bilateral lungs with large bilateral upper lobe cavitations. Labs were significant for leukocytosis with a left shift (white blood count (WBC) as 21.8 x10(3)/mcL with 81% neutrophils), microcytic anemia (hemoglobin was 8.2 with a mean corpuscular volume of 76), elevated C-reactive protein (CRP) at 23.8 mg/dl. Influenza B was found to be positive.

Due to the complexity of the presenting case, the patient was transferred to an outside larger institution. Sputum sample was positive for acid-fast bacilli (AFB). Treatment for suspected Mycobacterium TB was initiated with high dose isoniazid (INH), ethambutol, rifampin, levofloxacin, steroids, and oseltamivir. Due to amenorrhea and the patient’s failure to thrive, further imaging was obtained. Abdominal and pelvic CT showed calcified granulomas in the spleen and liver, a cystic mass in the right adnexa, and thickening of the ileum; findings indicative of disseminated TB. On hospital day four, the patient exhibited sluggish pupillary responses on physical exam. A brain magnetic resonance imaging (MRI) was obtained and revealed mild ventriculomegaly, thickening of the meninges, and focal areas of demyelination in the left parietal and left frontal lobes, consistent with central nervous system (CNS) TB. The anisocoria spontaneously resolved 24 hours later.

Sputum cultures ultimately demonstrated high resistance to INH (>2) and low resistance to streptomycin (>32). Despite this resistance, INH was continued due to its ability to penetrate the blood-brain barrier to target the CNS involvement. With continued treatment, her AFB smears and cultures were found to be negative in the blood, urine, cerebrospinal fluid, and bone marrow.

Throughout her hospital stay, the patient developed worsening hypoxia, requiring 2L of oxygen via nasal cannula nightly, she spiked daily fevers (Tmax 101F). Due to poor weight gain, she was transferred to our institution, with a specialized pediatric hospital, for medical and nutritional optimization. On admission, her albumin level equaled 3.4 g/dl (normal range for 16 years old is 3.9 to 5.1 g/dl) and her complete blood count (CBC) was 17.1 x10(3)/mcL, with a neutrophil percentage of 84.6 and a lymphocyte percentage of 9.4. She was managed with cyproheptadine and a nasogastric tube for overnight feeds in order to maximize caloric intake. The patient successfully gained 5 kg over three weeks and was subsequently discharged home on an appetite stimulant and to complete antimicrobial treatment.

Of note, the patient’s household members were also tested for TB. The patient’s mother was diagnosed with latent TB, the 9-year-old brother was diagnosed with active TB, and the mother’s live-in boyfriend was negative for TB.

## Discussion

Disseminated TB is a rare manifestation of TB infection. When it does arise in children, it is generally limited to infants with an underdeveloped immune system and in immunosuppressed children, such as those with co-morbid HIV infections [4]. The case presented here is unique in that the patient previously was an immunocompetent adolescent who developed widely disseminated TB. Our investigation focused on why a healthy adolescent female might develop disseminated TB.

Studies have suggested that hormonal changes around the time of puberty may influence the pathogenesis of active TB in the pediatric population [5-6]. Estrogen has been shown in vitro to drive the pro-inflammatory TH1-mediated immune responses [7-9], while testosterone has been found to inhibit these responses [10-12]. In a murine study conducted by Bini et al., the course of TB infection was compared between castrated mice and non-castrated mice. The study demonstrated that the castrated mice, with decreased androgen and testosterone levels, had a more favorable TB course than the non-castrated mice [12], which confirms the potential immunosuppressive effects of androgen hormones.

During puberty, sex hormone levels fluctuate greatly. In females, androgen concentrations, such as dehydroepiandrosterone (DHEA), rise prior to estrogens, and continue to rise until 20-24 years of age, before declining [13]. In addition to the immunosuppressive effects of androgens, alterations in the cortisol to DHEA ratio are associated with disturbances in concentrations of interferon-y, a key cytokine involved in TB containment [14]. In considering the many hormonal effects on the immune system, we gained insight into why our patient may have been at risk for a more serious TB infection and why she may have developed disseminated disease.

We also considered our patient's secondary amenorrhea and the potential hormonal role it played in her disseminated TB. Before becoming ill, her body mass index (BMI) was in the 37th percentile for age. Subsequently, she presented with a 9 kg weight loss, weighing 24 kg with a BMI below the first percentile for her age. It is possible that our patient’s hypothalamic hypogonadotropic amenorrhea (secondary to intentional malnutrition in an adolescent female versus the disease process itself) led to decreased follicle-stimulating hormone and luteinizing hormone, which led to decreased estrogen levels. In keeping with the hypothesized protective effect of estrogen, it is possible that our patient’s lack of estrogen may have resulted in the wide dissemination of her disease.

Additional considerations for our patient include the relationship between hypovitaminosis D and immunocompromise. Specifically, there is growing evidence that genes related to the metabolism of vitamin D contribute to TB susceptibility. The vitamin D receptor is found in many immune cells and is believed to play a role in cytokine secretion patterns, maturation of dendritic cells and T-cell function [15]. Upon transfer to our facility, our patient’s 25-hydroxyvitamin D level was 26.3 ng/mL. This insufficient vitamin D level could have contributed to her increased susceptibility to TB and the subsequent dissemination of the disease.

Lastly, In the setting of TB, nutritional status and the immune system has been linked in both the innate and adaptive immune system. Chronic malnutrition has been shown to decrease dendritic cell activity in mice, and as a consequence, T-cell activation [16]. With decreased T helper type 1 (Th1) activation, there is insufficient interleukin 12 (IL-12) and interferon gamma (IFN-gamma) to stimulate macrophages. A possible consequence of this sequence would be a failure of the host immune system to adequately contain the TB. Decreased T-cell activation has not previously been reported as the cause for disseminated TB. However, it remains possible that in the context of hormonal dysregulation, the additional insult of malnutrition may have been enough to precipitate immune insufficiency, and thus disseminated TB with CNS involvement.

## Conclusions

While we can only speculate as to the pathogenesis of dissemination, the case discussed above highlights the need for heightened awareness of TB among an otherwise healthy adolescent population. We suggest how pubertal changes may affect the risk of TB and the progression of disease. This heightened knowledge of the role that hormones play in the susceptibility of disseminated TB disease, in an otherwise healthy adolescent female, can help guide recognition and treatment of the disease to decrease morbidity and mortality.
